# Mechanical Mapping of the Common Carotid Artery in Healthy Individuals Aged 2 to 40 Years

**DOI:** 10.3390/jcm13206220

**Published:** 2024-10-18

**Authors:** Roch Listz Maurice, Nagib Dahdah

**Affiliations:** 1 Centre de Recherche Charles-Le Moyne (CRCLM), Centre Intégré de Santé et des Services Sociaux de la Montérégie-Centre (CISSSMC), Longueuil, QC J4K 0A8, Canada; 2 Groupe Biomédical Montérégie, Centre Intégré de Santé et des Services Sociaux de la Montérégie-Centre (CISSSMC), Brossard, QC J4V 2H1, Canada; 3 Service de Cardiologie, Département de Pédiatrie, Centre de Recherche du Centre Hospitalier Universitaire Sainte-Justine (CRCHUSJ), Montréal, QC H3T 1C5, Canada; nagib.dahdah.med@ssss.gouv.qc.ca

**Keywords:** common carotid artery, arterial wall stiffness, elastic modulus, stroke, ultrasound elastography, atherosclerosis, arteriopathy, imaging-based biomarker (ImBioMark), preventive medicine, medicine of the future

## Abstract

**(1) Background**: In 2022, the World Stroke Organization said there were more than 12.2 million new cases of stroke each year, between all ages and sexes. Six and a half million people die each year from stroke. Ischemic stroke accounts for 7.6 million (62%) cases, with 3.3 million (51%) deaths. Stroke is mainly linked to the atherosclerosis of a large artery. **(2) Objective**: Since the carotid artery directly supplies the brain, we used age-dependent mechanical mapping on the healthy common carotid artery (CCA) with the aim of being able to predict and thus potentially prevent ischemic stroke. **(3) Methods**: We assessed the CCA stiffness of 95 healthy control (CTL) females (2.23–39.46 years) and 107 healthy CTL males (2.85–40 years). Cine-loops of B-mode CCA data were digitally recorded with conventional medical ultrasound devices. Arterial wall elastic moduli were estimated offline using a proprietary non-invasive imaging-based biomarker algorithm (ImBioMark). Statistical analyzes were carried out with Excel software. **(4) Results**: Females showed a linear regression profile of CCA elastic moduli ranging from 41 ± 2 kPa to 54 ± 17 kPa (R^2^ = 0.88), while males showed one ranging from 38 ± 5 kPa to 63 ± 22 kPa (R^2^ = 0.83). For qualitative and quantitative illustrations, the elastic modulus data of CTLs were compared with those of subjects with Kawasaki disease and subjects born prematurely, respectively. **(5) Conclusions**: This study introduced some fundamental features of the mechanical evolution of the CCA as a function of age (2–40 years). Since atherosclerotic arteriopathy starts early in life, this gives the ability to predict risks of stroke and other cardiovascular diseases with the possibility of applying a more comprehensive range of potential preventive measures early in life. This is consistent with preventive medicine objectives which aim to be more predictive to implement pre-emptive measures as opposed to diagnostic and curative approaches.

## 1. Introduction

Cerebrovascular accident (CVA), also known as brain attack or stroke, is a medical condition in which poor blood flow supply to the brain causes cell death and subsequent brain dysfunction [1]. The effects of a stroke depend on what part of the brain has been damaged and how much damage has been done [1]. There are two main types of strokes, namely ischemic stroke due to obstruction to blood flow and hemorrhagic stroke due to blood loss and compartmental compression [1]. In both situations, the supply of oxygen and nutrients to the brain tissues is hindered.

In 2022, the World Stroke Organization (WSO) states that there are more than 12.2 million new cases of stroke each year, between all ages and both sexes. Six and a half-million people die from stroke annually. Ischemic stroke accounts for 7.6 million (62%) cases, with 3.3 million (51%) deaths [2]. Stroke is primarily related to the atherosclerosis of a large artery.

Atherosclerosis is characterized by the development of lesions in the walls of the arteries. These lesions can lead to the narrowing of the arterial walls due to the accumulation of atherosclerotic plaques [3]. The process of the development of atheromatous plaques, called atherogenesis, is characterized mechanically by the remodeling of the arteries [4].

Arterial remodeling is known to be strongly associated with changes in arterial stiffness [5]. Additionally, arterial stiffness itself increases with age [6]. This work quantifies the intrinsic mechanical remodeling of the common carotid artery (CCA) with age, in healthy control individuals (CTL). The aim is to be able to detect at an early stage any abnormal mechanical alteration in the CCA wall that could cause a stroke in the medium or long term and thus apply appropriate prophylactic measures.

## 2. Materials

In a very recent review addressing non-invasive cardiovascular risk factors assessment by Trimarchi et al. [7], the authors developed on the utility of various non-invasive biomarker techniques (Supra-Aortic Trunks Ultrasound, Arterial Stiffness and Pulse Wave Velocity, Echocardiography, Coronary Artery Calcium Score, Coronary Computed Tomography Angiography, Computer Tomography-Derived Coronary Flow Fractional Reserve, cardiac Magnetic Resonance imaging, and the most recent Photon-Counting CT). Every technique brings specific advantages despite inherent limitations, but a combination of techniques for any particular population disease needs represents reliable solutions to consider.

Since arterial stiffness is known to be an independent predictor of cardiovascular disease [6,8], non-invasive ultrasound vascular elastography (NIVE) was then proposed as a method to quantify the stiffness of superficial arteries [9,10,11]. While these previous applications used radio frequency data, NIVE was adapted to process B-mode images, a proprietary referred to as ImBioMark (Imaging-based BioMarker) [12]. For reference, ImBioMark has already been adapted to assess ascending aortic wall remodeling in adolescents with Kawasaki disease [12] and to study carotid health status in adolescents born with intrauterine growth restriction [13]. For the present study, statistical analyzes (regression, *t*-test (*p* < 0.05), correlation, mean ± std, etc.) were carried out with Excel software (Microsoft^®^ Excel^®^ 2013 (15.0.5579.1001)).

## 3. Methods

### 3.1. The Control Population Studied

As illustrated in Table 1, the control population studied (CTL) is composed of 95 females (from 2.23 to 39.46 years) and 107 males (from 2.85 to 40 years), for a total of 202 CTL subjects. For the purposes of these analyses, the female and male populations are subdivided into age subgroups as follows:

(1) $${⋃}_{n}\;]\begin{array}{cc} n & n+5 \end{array}],\;n\in \;\left\{0,\;5,\;10,\;\ldots ,\;35\right\}$$

CTL subjects were recruited as part of several research studies, including the assessment of vascular health following Kawasaki disease, assessment of vascular function in essential pediatric hypertensive patients, and vascular monitoring of children born prematurely. Exclusion criteria were pregnancy (adult females), diabetes, known atherosclerotic cardiovascular diseases, treatment for hypertension and/or hyperlipidemia, or chronic inflammatory conditions requiring previous or ongoing treatment. In summary, all CTL subjects were free of known cardiovascular comorbidities or known cardiovascular risk factors. The institutional ethics committee approved the studies (The Sainte-Justine UHC REB (#FWA00021692); #2017-1411—9 June 2017; #2016-1124–1 April 2017; #2014-3901—7 July 2014), and written informed consent was obtained from adult subjects and parents of minor subjects for each study.

### 3.2. Subjects in Pathological Conditions

These subjects were recruited as part of two research studies, respectively, investigating the assessment of vascular health after Kawasaki disease and the assessment of vascular function in children born prematurely. Exclusion criteria, institutional review board approval, and written informed consent were exactly the same as those reported above for CTL subjects.

### 3.3. Data Acquisitions

Weight and height were obtained, and blood pressure was measured with typical automated sphygmomanometers used in medical settings. B-mode data from longitudinal segments of the generally straight CCA were recorded with typical commercial ultrasound devices used in medical settings, using probes in the range of 8 to 12 MHz. The frame rate was generally between 30 and 40 Hz depending on the depth, which was generally 4 cm with the focus positioned in the middle of the CCA. Loops of 7 to 8 beats were recorded in series. Electrocardiography (ECG) signals were recorded simultaneously for the proper determination of the cardiac cycle as well as the correct identification of systole and diastole.

### 3.4. ImBioMark and Elastic Modulus Calculation

**Figure 1:**
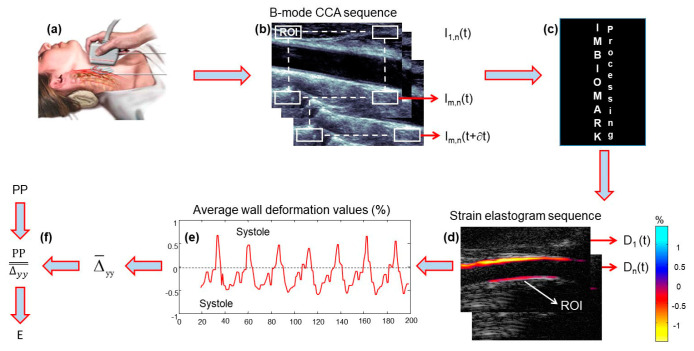
Reminder of the main steps of ImBioMark. (**a**,**b**) B-mode CCA ultrasound data sequences $I\left(t\right)$ recorded non-invasively; (**c**) ImBioMark processing algorithm computes axial strain elastograms; (**d**) Illustration of an axial strain elastogram $D_{n}\left(t\right)$; (**e**) Illustration of a CCA deformation curve; (**f**) Calculation of ImBioMark elastic modulus (E). The colorbar in (**d**) quantifies the deformation in (%). E, the elastic modulus in (**f**), is expressed in kPa.

ImBioMark (Imaging-based BioMarker) has been extensively described, tested, and discussed elsewhere [12,13,14]. Only for the purposes of illustration, the diagram in Figure 1 briefly describes the main steps of ImBioMark. The B-mode CCA ultrasound data sequences $I\left(t\right)$ were recorded non-invasively (Figure 1a,b). $I\left(t\right)$ was divided into sub-regions of interest (ROIs), i.e., $I_{\left(m,n\right)}(t)$, which were used as input to the ImBioMark processing algorithm (Figure 1c). ImBioMark then computed the axial deformation of the CCA wall ($\Delta_{yy}\left(t\right)$) for each ROI to provide sequences of axial deformation elastograms ($D_{n}\left(t\right)$) in Figure 1d. For each ($D_{n}\left(t\right)$), the mean of $\Delta_{yy}\left(t\right)$ was calculated for the bottom wall to provide the CCA deformation curve in Figure 1e.

To complete the estimation process, cumulated systolic (${\bar{\Delta }}_{yy}$^syst^) and cumulated diastolic (${\bar{\Delta }}_{yy}$^diast^) deformations, respectively, were averaged over at least three cardiac cycles. ${\bar{\Delta }}_{yy}$ was then calculated as the average in absolute values of ${\bar{\Delta }}_{yy}$^syst^ and ${\bar{\Delta }}_{yy}$^diast^. ImBioMark elastic modulus (E), for a given subject’s CCA, was calculated as the ratio between the pulse pressure (PP ≡ peak-systole blood pressure − nadir-diastole blood pressure) and ${\bar{\Delta }}_{yy}$ (Figure 1f).

(2) $$E=\frac{PP}{{\bar{∆}}_{yy}}$$

## 4. Results

### 4.1. Somatic Data

**Figure 2:**
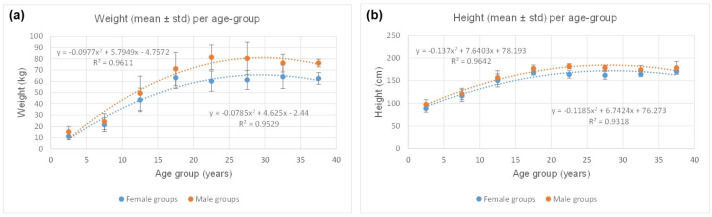
(**a**) Quadratic polynomial regressions showing weight stability at 61 ± 8 kg (R^2^ = 0.95) and 80 ± 11 kg (R^2^ = 0.96) for females and males, respectively; (**b**) height stability is observed at 165 ± 8 cm (R^2^ = 0.93) and 179 ± 8 cm (R^2^ = 0.96) for females and males, respectively. For both genders, somatic stability is reached around the age of 25.

Table 2 contrasts females’ and males’ somatic data. As shown in Figure 2, the curves for females are consistent with quadratic polynomial regressions, indicating weight stability at 61 ± 8 kg (R^2^ = 0.95) and height stability at 165 ± 8 cm (R^2^ = 0.93) around age 25. The profiles are roughly the same for males but showing weight stability at 80 ± 11 kg (R^2^ = 0.96) and height stability at 179 ± 8 cm (R^2^ = 0.96) around the same age. Additionally, males are observed to be heavier (*p* = 0.0015) and taller (*p* = 0.0003) than females for each age group.

### 4.2. Physiological Data

**Figure 3:**
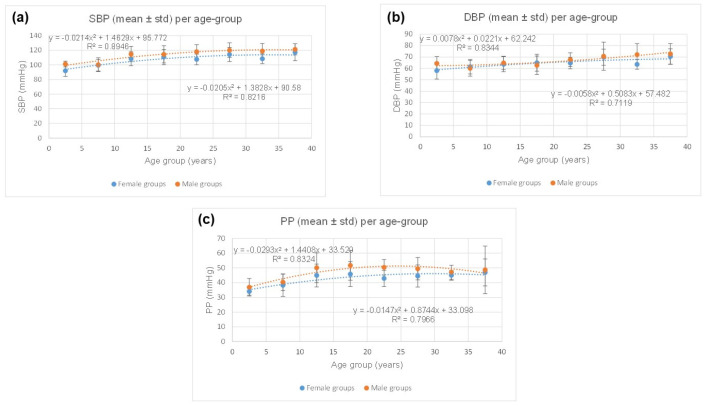
Contrasting females’ and males’ physiological data. Males’ SBP (*p* = 0.0007) and PP (*p* = 0.0006) were observed higher than those for females for each age group, although no statistically significant difference was observed for DBP (*p* = 0.0580).

Table 3 contrasts females’ and males’ physiological data. The ranges of systolic blood pressure (SBP), diastolic blood pressure (DBP), and pulse pressure (PP) values are consistent with those expected for healthy females and males [15,16]. As shown in Figure 3, for SBP, DBP, and PP, the plots are consistent with quadratic polynomial regressions with R^2^, ranging from 0.71 to 0.89 for females and males. Additionally, males’ SBP (*p* = 0.0007) and PP (*p* = 0.0006) are observed to be higher than those for females for each age group, although no statistically significant difference is observed for DBP (*p* = 0.0580).

### 4.3. CCA Stiffness According to Age

The two-tailed observational *t*-test showed significant somatic differences between females and males, particularly in terms of weight (*p* = 0.0002) and height (*p* = 0.00005), observed between 10 and 15 years; this was not significantly associated with changes in CCA mechanics (*p* = 0.4376). Consequently, the mechanical data were pooled for females and males in the age groups of [0–5] and [10–15], respectively.

Table 4 contrasts the elastic moduli (E) of the CCA between CTL females and CTL males, as a function of age. It is observed that below the age of 20, there is no statistical difference between the genders (*p* = 0.1439). In contrast, beyond the age of 20, males’ CCAs are quantified as being more rigid (*p* = 0.0102).

**Figure 4:**
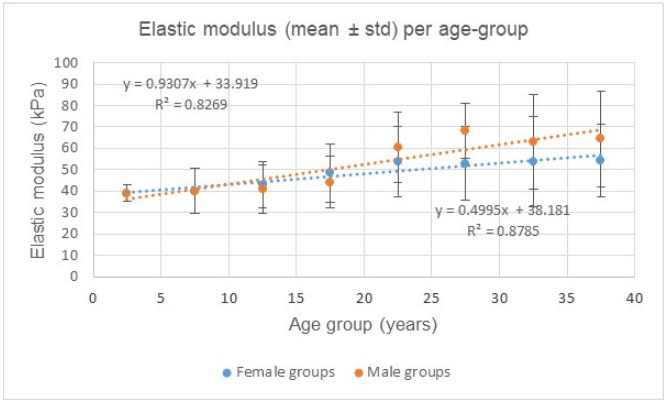
Elastic moduli (E) for female and male CTL subjects as a function of age groups. Males’ CCA plot has a slope (0.9307) almost twice as steep as females’ slope (0.4995), indicating that males’ CCA increases more rapidly with age than females.

Figure 4 plots the elastic moduli for female and male CTL subjects as a function of age. In both cases, the mechanical evolution of the CCA with age is consistent with a crescendo linear regression (R^2^ > 0.82). Males’ CCA plot has a slope (0.9307) almost twice as steep as females’ slope (0.4995), indicating that males’ CCA stiffness increases more rapidly with age than females’.

### 4.4. Contrasting Mechanical Data and Somatic Data

**Figure 5:**
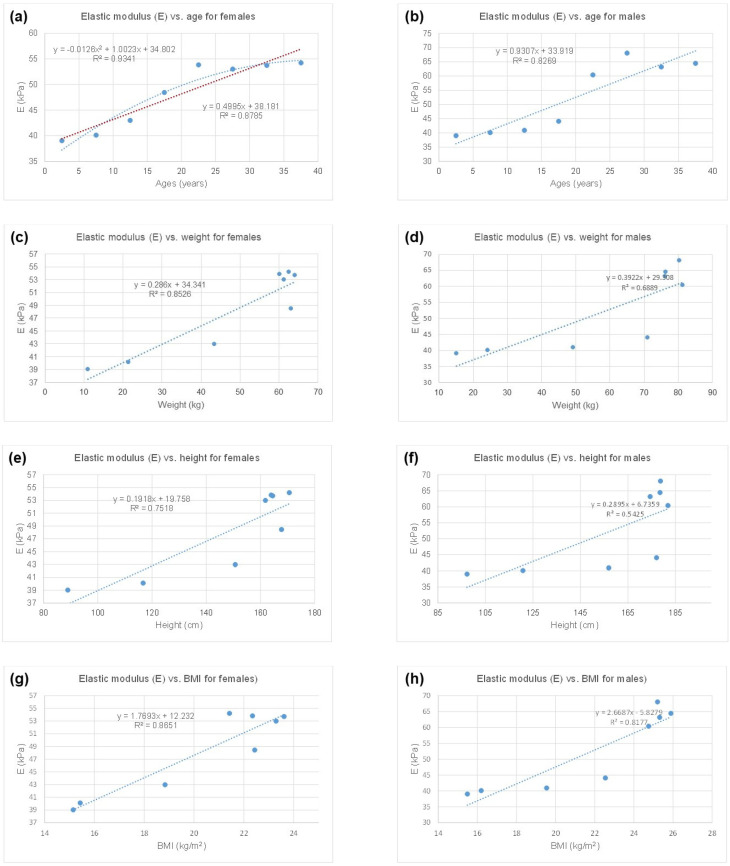
Contrasting mechanical and somatic data, with respect to gender. (**a**) Elastic modulus (E) vs. age, for females; (**b**) elastic modulus (E) vs. age, for males; (**c**) elastic modulus (E) vs. weight, for females; (**d**) elastic modulus (E) vs. weight, for males; (**e**) elastic modulus (E) vs. height, for females; (**f**) elastic modulus (E) vs. height, for males; (**g**) elastic modulus (E) vs. BMI, for females; (**h**) elastic modulus (E) vs. BMI, for males.

In Figure 5, we contrast mechanical and somatic data, with respect to gender. As shown in Figure 5a, the evolution of the CCA stiffness according to age, for females, seems to be optimally represented by a quadratic polynomial regression (R^2^ = 0.9341). Nevertheless, the linear regressions are equivalently consistent with age (Figure 5a; R^2^ = 0.8785), weight (Figure 5c; R^2^ = 0.8526), and BMI (Figure 5g, R^2^ = 0.8651); with BMI (body mass index) being calculated as follows: Weight (in kg)/(height (in m))^2^. Furthermore, for males, the best linear regression consistencies between mechanical and somatic data are provided by age (Figure 5b; R^2^ = 0.8269) and BMI (Figure 5h; R^2^ = 0.8177).

### 4.5. Comparison of CCA Stiffness of CTL Subjects with That of Subjects in Pathological Conditions

Table 5 presents the populations in pathological conditions which were studied to compare the CTL population. In particular, subjects born prematurely (PMB) and subjects with Kawasaki disease (KD) were, respectively, included in the validation part of this study. As mentioned above, these data were mostly part of some previous papers [12,13,14] on the topic of mechanical properties of CCA. For the purpose of this study, we only consider KD subjects who did not develop a coronary aneurysm. In summary, 38 females and 39 males, aged 5 to 30 years old, were included.

**Figure 6:**
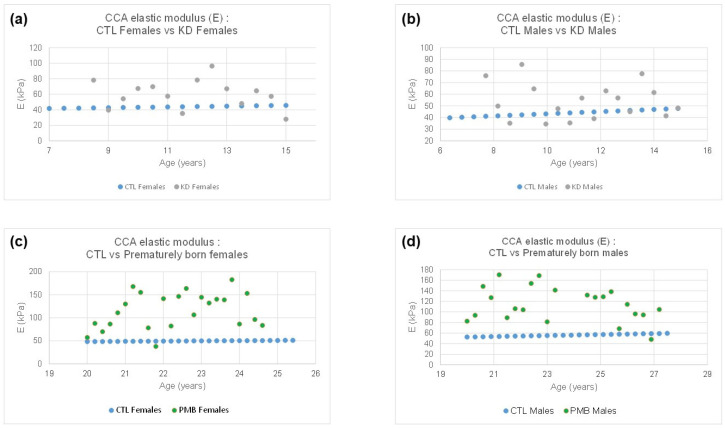
Comparing the reference (CTL) CCA elastic moduli (E) with those obtained in KD and PMB for female (**a**,**c**) and male (**b**,**d**) subjects.

Figure 6 compares the reference (CTL, “●”) CCA elastic moduli (E) with those obtained in KD (“●”) and PMB (“●”) subjects. It is worth recalling that, as shown in Figure 4, the expected CCA E, as a function of age, are given for females (3) and males (4) as follows:

(3) y=0.4995x+38.18

(4) y=0.9307x+33.92

with “x” being the age of the subject.

Figure 6a,b shows that the CCA of KD (“●”) subjects tends to be significantly stiffer in both females (a) and males (b) than in CTL (“●”); *p* = 0.0069 and *p* = 0.0258, respectively. Figure 6c,d compares the reference (CTL, “●”) CCA elastic modulus (E) with that obtained in premature born (PMB, “●”) subjects. In both cases, females (c) and males (d), the CCA of PMB subjects tends to be much more rigid than that in CTL (*p* < 10^−7^).

## 5. Discussion

### 5.1. Summary of Main Results

The main result of this study is the introduction of age-dependent mechanical mapping of the healthy common carotid artery (CCA). Although the data reported here mainly relate to the mechanics of the CCA in children, adolescents, and young adults (≤40 years), to our knowledge, this is the first time that such mapping of the artery mechanics is proposed in the literature, specifically CCA-ImBioMark-MAP.

It is important to note that CCA-ImBioMark-MAP is not a clinical diagnostic tool. At this stage, we suggest comparative studies including pathological cases in comparison to the healthy CTL subjects. In the same line of thought, studying a series of pathological cases before and after response to specific treatments could be another step towards prognostication via the stage of determining the variation in ImBioMark with other effective therapeutic means. Eventually, the primary use of ImBioMark should be in the monitoring of preventive interventions, the monitoring of therapeutic response, and hopefully in the assessment of the prognostic value of early detection of dysfunction of mechanical alteration in the artery at an early stage of a disease.

Typical qualitative and quantitative examples of the application of CCA-ImBioMark-MAP are reported above for subjects with Kawasaki disease (KD) and born prematurely (PMB). In KD and PMB subjects, the CCA is reported to be significantly stiffer than that of controls (CTL); this indicates that atherosclerosis-like mechanisms likely occur in CCA of these patients; then, preventive measures should be applied to minimize the potential risks of cardiovascular diseases.

### 5.2. Advantages of ImBioMark

The current database of controls demonstrates a narrow variance in ImBioMark measurements around the average. This feature is in favor of the technique, which permits statistically distinguishable differences from pathological states, such as Kawasaki disease and premature born babies. The fact that the elastic modulus equations progress in a linear fashion compared to age, both in males and in females, renders the calculation of an equation for standard deviation derivatives rather simple. The use of standard deviation is currently considered a gold standard to compare biomarkers in children. Measurements that deviate beyond the second standard deviation (outside the boundaries delineated between −2 and +2 Z-score) are considered abnormal. The farther it is from the second standard deviation, the more pathological the situation.

### 5.3. Comparisons Between Males’ and Females’ Data

Our data demonstrated higher elastic modulus values in older males compared to older females. The deviation between sexes onsets around adolescence and continues to deviate later on (Figure 4). Not surprisingly, adult and teenage males exhibit higher blood pressure compared to females. In addition, atherosclerotic vascular disease is also closely linked to male hormones (testosterone), not to mention the risk of cardiovascular disease in general, some of which is likely linked to sex chromosome interactions.

There is evidence that biological sex interferes with cardiovascular outcomes, starting from early childhood, and this is not exclusively dependent on sex hormonal influence. For example, biological sex influences Kawasaki disease, predominantly of a pre-pubertal onset, with males affected 50% more compared to females [17]. Additionally, Kawasaki disease male patients are more prone to developing coronary artery complications, with a risk of coronary stenosis and death from cardiac events compared to females [18,19]. The standardized mortality rate of males with coronary artery sequelae secondary to Kawasaki disease is three-fold that of females according to the Japanese nationwide literature [20].

### 5.4. Limitations of the Study

The main weakness of this study could be the relatively small sample of the control population studied, specifically aged under 5 and over 25 years. However, this issue was partly resolved, for the young population, by pooling the CCA data of females and males for [0–5] and [10–15] age groups, respectively.

It is thought that increasing the sample size of CTL subjects older than 25 years may slightly alter the overall crescendo of CCA stiffness with age. Furthermore, it would be interesting to further study the population over 40 years of age in order to be able to quantify the maximum range of stiffness potentially achievable for the CCA of healthy subjects.

## 6. Conclusions

This study made it possible to introduce some fundamental features of the mechanical evolution of the carotid artery as a function of age and somatic data; it is CCA-ImBioMark-MAP. Supported and validated by clinical data, the potential of ImBioMark-MAP has been evaluated and demonstrated.

Since atherosclerotic arteriopathy starts early in life, the age range values (children to young adults) we provide in this study gives the ability to predict risks of ischemic stroke and other cardiovascular diseases with the possibility of applying a more comprehensive range of potential preventive measures early in life as variations may be detectable. In summary, ImBioMark-MAP aims to determine an advanced measurable risk facture for preventive intervention purposes before irreversible formation occurs. This is consistent with preventive medicine objectives which aim to be more predictive to implement pre-emptive measures as opposed to diagnostic and curative approaches.

## Figures and Tables

**Table 1:** The studied control population (CTL) is aged 2.23 to 40 years. For the purposes of these analyses, the female and male populations were subdivided into age subgroups ranging from [0–5] to [35–40] years.

Population Investigated (n = 95 Females + 107 Males)								
Age Group (years)	[0–5]	[5–10]	[10–15]	[15–20]	[20–25]	[25–30]	[30–35]	[35–40]
Nb of Females	5	18	18	15	20	8	5	6
Nb of Males	7	30	29	10	11	9	5	6

**Table 2:** Contrasting females’ and males’ somatic data. Males are observed heavier (*p* = 0.0015) and taller (*p* = 0.0003) than females for each age group.

	Females		Males	
Age Group	Weight (kg) Mean ± Std	Height (cm) Mean ± Std	Weight (kg) Mean ± Std	Height (cm) Mean ± Std
[0–5]	11 ± 3	88 ± 9	15 ± 5	97 ± 11
[5–10]	21 ± 6	116 ± 13	24 ± 7	120 ± 13
[10–15]	43 ± 10	150 ± 14	49 ± 15	156 ± 15
[15–20]	63 ± 10	167 ± 5	71 ± 15	177 ± 8
[20–25]	60 ± 9	163 ± 8	81 ± 11	181 ± 6
[25–30]	61 ± 8	161 ± 9	80 ± 14	178 ± 6
[30–35]	64 ± 10	164 ± 5	76 ± 8	174 ± 9
[35–40]	62 ± 5	170 ± 4	76 ± 3	178 ± 14

**Table 3:** Contrasting females’ and males’ physiological data. The ranges of SBP, DBP, and PP values are consistent with those expected for healthy females and males [15,16].

	Females			Males		
Age Group	SBP (mmHg) Mean ± Std	DBP (mmHg) Mean ± Std	PP (mmHg) Mean ± Std	SBP (mmHg) Mean ± Std	DBP (mmHg) Mean ± Std	PP (mmHg) Mean ± Std
[0–5]	92 ± 8	58 ± 7	34 ± 3	101 ± 4	64 ± 6	36 ± 6
[5–10]	99 ± 8	61 ± 6	38 ± 7	100 ± 9	60 ± 7	40 ± 6
[10–15]	108 ± 10	63 ± 7	44 ± 8	114 ± 11	64 ± 6	50 ± 10
[15–20]	110 ± 10	64 ± 7	45 ± 8	114 ± 12	62 ± 8	51 ± 10
[20–25]	107 ± 7	64 ± 5	42 ± 5	118 ± 10	67 ± 6	50 ± 5
[25–30]	114 ± 10	69 ± 7	44 ± 7	120 ± 10	70 ± 12	49 ± 8
[30–35]	108 ± 7	63 ± 4	45 ± 3	119 ± 10	72 ± 10	47 ± 5
[35–40]	117 ± 12	70 ± 7	46 ± 9	121 ± 8	72 ± 9	48 ± 16

**Table 4:** Contrasting CCA stiffness in females and males, as a function of age. Beyond the age of 20, the CCAs of males were observed to be more rigid than those of women (*p* = 0.0102).

Population Investigated after Data Pooling (n = 132 Females + 130 Males) The Values of the Elastic Modulus (E) are Expressed in kPa								
Age-group (years)	[0–5]	[5–10]	[10–15]	[15–20]	[20–25]	[25–30]	[30–35]	[35–40]
Nb of Females	12	48	18	15	20	8	5	6
Nb of Males	12	48	29	10	11	9	5	6
E (mean ± std) Females	39 ± 4	40 ± 10	43 ± 11	48 ± 13	54 ± 16	53 ± 17	54 ± 21	54 ± 17
E (mean ± std) Males	39 ± 4	40 ± 10	40 ± 11	44 ± 12	60 ± 17	68 ± 13	63 ± 22	64 ± 22

**Table 5:** Portrait of the populations in the pathological conditions studied. CCA elastic moduli for these subjects have mostly been evaluated elsewhere [12,13,14] but reported in this article to compare the CTL population.

Population of Individuals in Pathological Conditions Investigated (n = 38 Females + 39 Males)								
Age Group (years)		[0–5]	[5–10]	[10–15]	[15–20]	[20–25]	[25–30]	Total
Prematurely Birth (PMB)	Nb females					24		24
	Nb males					12	10	22
Kawasaki Disease (KD)	Nb females		4	10				14
	Nb males		7	10				17

## Data Availability

The authors declare data availability.
